# Cultivation of Entrepreneurial Psychological Quality and Optimization of Piano Talents Training in Colleges and Universities Through Questionnaire Survey

**DOI:** 10.3389/fpsyg.2022.844231

**Published:** 2022-06-24

**Authors:** Yinai Gao

**Affiliations:** Music College, Shandong Normal University, Jinan, China

**Keywords:** college piano talents, entrepreneurial psychological quality, innovative thinking, risk challenging, questionnaire survey

## Abstract

The mechanism is studied to optimize the cultivation of piano talents and entrepreneurial psychological quality (PSYQ) in colleges and universities through the QS (Questionnaire Survey). Firstly, the cultivation of piano talents’ entrepreneurial consciousness is explored, together with the entrepreneurial will, entrepreneurial personality, and entrepreneurial ability. Secondly, the piano talents entrepreneurial PSYQ training model is established according to the internal factors of college students majoring in piano, entrepreneurial attitude, students’ interpersonal network, and entrepreneurial environment factors. The correlations between various variables are analyzed. Afterward, a QS is designed, and the data are collected from university A in the city of Xi’an. Data analysis shows significant differences in the cultivation of entrepreneurial PSYQ among students of different grades in college Piano Majors, namely, *p* < 0.01. Students growing up in different environments show significant differences in entrepreneurial PSYQ, namely, *p* < 0.01. Meanwhile, whether students have served as cadres will also have a particular impact on students’ entrepreneurial PSYQ, namely, *p* < 0.01. However, the dimension of only-child-or-not in entrepreneurial PSYQ training does not show a significant difference. Finally, under the model analysis, achievement motivation, entrepreneurial attitude, entrepreneurial interest, entrepreneurial consciousness, interpersonal network, entrepreneurial environment, and personal entrepreneurial background will affect the cultivation of their entrepreneurial PSYQ. They show a positive correlation with the entrepreneurial PSYQ. Therefore, in the future college piano talents training and entrepreneurial PSYQ training, more focus should be put on students’ innovative thinking and risk challenging capacity, thereby helping students with better entrepreneurial work.

## Introduction

With the proposal of the development strategies of “Improving the Ability of Independent Innovation and Building an Innovative Country” and “Promoting Entrepreneurship to Drive Employment”, and “Mass Entrepreneurship and Innovation” as the new engine of China’s economy, innovation and entrepreneurship have risen as a national development strategy in recent years (Yan et al., 2018). In building a national innovation system and creating an innovation and entrepreneurship atmosphere, college students’ Innovation and Entrepreneurship Education (IEE) is essential. Innovation and Entrepreneurship Education can train innovative and entrepreneurship talents to drive and promote the innovation and entrepreneurship activities of the whole society (Ramchander, 2019; Isabelle, 2020). In particular, colleges and universities (CAU) can help students establish the spirit of exploring and seeking knowledge by cultivating college students’ basic innovation and entrepreneurship quality. Such a spirit can lead college students to continuously tap their potential, stimulate their enthusiasm for innovation and creation, discover new markets, and develop new technologies, energy, and processes to start business and social welfare entrepreneurship in the future. The performance of on-the-job entrepreneurship is more active, providing better products and services for society (Du et al., 2020).

This paper studies how to use the Questionnaire Survey (QS) to optimize the cultivation of piano talents and entrepreneurial Psychological Quality (PSYQ) in CAU. Then, it takes the college Piano Majors as the research object, collects data by the designed QS, analyzes the data through Structural Equation Modeling (SEM), and studies the relevant influencing factors. It is hoped that the present work can help CAU actively cultivate the entrepreneurial PSYQ of Piano Majors to encourage them to grow into excellent social talents. The innovation point of the present work is establishing the SEM to analyze various factors of students’ entrepreneurial PSYQ, thereby obtaining high accuracy and enhancing the persuasiveness of the conclusion.

## Related Work

The present work aims to help college students cope with rapid social development and improve their independent innovation ability. Concerning such aspects, many research teams investigate improving the entrepreneurial PSYQ of college Piano Majors. Wu and Wu (2017) found that Entrepreneurship Education (EE) research in the Asia Pacific region was relatively limited in research plan, views, and levels. Based on the discussion of key topics, it was suggested to conduct a multi-faceted, multi-level perspective and vertical research to have a broader and deeper understanding of the complexity of EE and its role in the relationship with national entrepreneurship. Meanwhile, the research bridged the gap between Western and Asian research backgrounds and continuously developed a standard knowledge system (Wu and Wu, 2017). Wang S. et al. (2019) discovered that the innovation and entrepreneurship plan positively impacted all entrepreneurial abilities and entrepreneurial intentions. The effect of attitude was significantly better than that of knowledge or skills, and academic disciplines did have a situational impact on students’ entrepreneurial abilities and entrepreneurial intentions (Wang S. et al., 2019). Wu and Mao (2020) analyzed college students’ views on the relationship between entrepreneurial environment perception and entrepreneurial motivation. The results showed that college students’ entrepreneurial motivation was significantly and positively affected by their perception of socio-economic status, education, training, and financial and non-financial support (Wu and Mao, 2020). Sang and Lin (2019) unveiled that entrepreneurial alertness played a significant mediating and regulating role in entrepreneurship by studying the relationship between College Students’ entrepreneurship education, entrepreneurial alertness, and entrepreneurial intention. It was of great significance for EE to explore and study the role of entrepreneurial alertness in the process of stimulating entrepreneurial intention (Sang and Lin, 2019). Suacamram (2019) revealed that visiting museums and Broadway shows could stimulate creativity and inspiration by studying the differences between student groups in cultivating creativity and entrepreneurship. Teachers also played a role in fostering creativity and inspiration. Additionally, the students participating in the field trip had significantly improved their creativity and entrepreneurship (Suacamram, 2019).

According to the above literature, the research on IEE in China is still in its infancy. Although there is some related research, they still have some limitations. Mainly because the content of college IEE is pervasive, and the cultivation methods of students’ PSYQ vary significantly according to CAU.

## Research Scheme Design

### Cultivation of Entrepreneurial Psychological Quality

#### Cultivation of Entrepreneurial Consciousness of Piano Talents

Usually, entrepreneurial consciousness refers to the personality consciousness that encourages and promotes entrepreneurs during entrepreneurial activities, including the entrepreneurial needs, responsibility consciousness, interest, motivation, ideal, beliefs, and world outlook (Wang S. et al., 2019). In the first place, entrepreneurial consciousness is guided by entrepreneurship. Before actual entrepreneurial behaviors are performed, entrepreneurial consciousness should be clarified and stiffened, and as the core power of entrepreneurs, entrepreneurial consciousness is the main reason for entrepreneurs’ persistence, as well as the psychological motivation of entrepreneurial success tendency (Ghafar, 2020). Sound entrepreneurial consciousness is manifested in many aspects, such as a strong sense of responsibility, clear entrepreneurial purpose, strong entrepreneurial motivation, self-confidence, strong entrepreneurial interest, firm belief, and lofty ideals (Wang S. M. et al., 2019).

#### Cultivation of Piano Talents’ Entrepreneurial Will

Commonly, entrepreneurial will refers to the PSYQ of entrepreneurs to overcome the difficulties during the entrepreneurial process and stick to the entrepreneurial actions, which play a regulatory role in entrepreneurial activities. For one thing, entrepreneurship can help launch or strengthen the necessary actions to achieve entrepreneurial purposes (Li and Wu, 2019). It can also stop or weaken actions contradicting the intended entrepreneurial goals. Inevitably, various difficulties may emerge throughout the entrepreneurial process, and apart from professional knowledge and external help, entrepreneurs have to rely on their firm will to overcome these difficulties (Garcia et al., 2019). Hence, a firm entrepreneurial will play a decisive role in entrepreneurship success. Usually, the entrepreneur will is subdivided into perseverance, fortitude, optimism, and courage.

#### The Cultivation of Piano Talents’ Entrepreneurial Personality

Entrepreneurial personality refers to the quality of an entrepreneur’s stable and distinctive entrepreneurial personality, a kind of individual character different from other people’s mental outlook. Entrepreneurial personality will profoundly impact entrepreneurs’ initiatives and ways of thinking during entrepreneurship and is the indispensable quality for successful entrepreneurship (Darmanto and Yuliari, 2018; Wang et al., 2018; Bloemen-Bekx et al., 2019). Unique entrepreneurial personality includes the spirit of adventure, the courage to go beyond the general people, open up new roads, and seize the market opportunities to create a truly vibrant cause. The spirit of innovation is to constantly innovate and surpass themselves during entrepreneurship, thereby improving their market competitiveness and the sustainable development of the industry and enterprises themselves. Independent decisiveness refers to the ability of entrepreneurs to solve and analyze problems independently, with which a particular decision can be carried out or specified. Meanwhile, independent decisiveness can reduce the influence of external factors on entrepreneurs and help them focus on their self-development (Meoli et al., 2020). Entrepreneurs should also have a sense of boldness, courage to assume responsibility, and a series of social, legal, economic, and other responsibilities that they should bear, which is the necessary quality of successful entrepreneurs. Meanwhile, entrepreneurs should have a positive working attitude and always maintain enthusiasm for entrepreneurial projects to enter the best entrepreneurial state. In every stage of entrepreneurship, a calm mentality is a key to solving the problem (Daniel et al., 2019; Guzman et al., 2020).

#### Cultivation of the Entrepreneurial Ability of Piano Talents

Generally, entrepreneurial ability refers to the solid, comprehensive psychological characteristics entrepreneurs must have to complete entrepreneurial activities. The entrepreneurial ability of entrepreneurs will have a direct impact on the efficiency of entrepreneurial practice and is also an essential psychological condition to help smooth entrepreneurship. Entrepreneurial ability is divided into knowledge application ability, keen insight and judgment, self-discipline ability, communication and cooperation ability, and market analysis control ability. It also involves adapting to the environment, management ability, foresee and risk-bearing ability, and self-regulation ability (Liu et al., 2019).

### Construction of Entrepreneurial Psychological Quality Training Model for Piano Talents

#### Internal Factors of College Piano Majors: Achievement Motivation, Entrepreneurial Interest, and Entrepreneurial Quality

Entrepreneurial interest refers to the degree of material and spiritual needs of college students majoring in piano for entrepreneurship, mainly manifested in the initiative, curiosity, and aspiration for knowledge of entrepreneurial activities (Kucher et al., 2019). Achievement motivation refers to the original motivation of college students majoring in piano to achieve entrepreneurial activities. For example, entrepreneurship can bring wealth, status, self-worth, and social contributions. Entrepreneurial quality refers to the unique personal characteristics of college students majoring in piano that affect entrepreneurial consciousness. Here, the research is specifically carried out from five aspects: risk-taking, risk-taking consciousness, will quality, innovative thinking, and difficulties overcome (Hutagalung et al., 2017).

#### Entrepreneurial Attitude of College Students Majoring in Piano

The entrepreneurial attitude of piano students refers to their preference for entrepreneurial activities. It has been argued that personal interest, achievement motivation, and trait quality impact entrepreneurial attitude. Among the entrepreneurial consciousness factors, entrepreneurial attitude is very important. Thus, it is necessary to study the relationship between entrepreneurial attitude and individual entrepreneurial interest, achievement motivation, and entrepreneurial quality, as well as the importance and influence mechanism of entrepreneurial attitude as an intermediary variable. Concerning the model simplicity, the entrepreneurial attitude studied here is not divided into subdimensions and is scientifically reflected through multiple items (Darmanto and Yuliari, 2018).

#### Interpersonal Network of Piano Majors in Colleges and Universities

As a relationship of interaction and connection in and between individuals and social groups, the interpersonal network has a particular impact on individual attitude, consciousness, and even behavior. Here, the interpersonal network of piano students in CAU may also impact entrepreneurial attitude and entrepreneurial consciousness (Apriana et al., 2019). The interpersonal network includes the network relationship between people and the role of individuals in a group. Therefore, the dimensions of an interpersonal network include individual networks and group networks. Additionally, the nodes in the interpersonal network are interconnected by various complex network relationships, which is another dimension of the interpersonal network (Jemal, 2017). Some scholars divide the social network into three dimensions: network strength, network scale, and heterogeneity. The proposed three dimensions here do not conflict with the research of existing scholars but have considered the special research object of piano students in CAU. Under the three dimensions of individual, group, and relationship, the network nature of piano students in CAU can be better reflected. Meanwhile, the proposed three dimensions cover interpersonal networks’ strength, scale, and heterogeneity.

#### The Entrepreneurial Environment Factors of College Students Majoring in Piano

Obviously, the theory of planned behavior suggests that behavior is caused by the perceptual control of attitude, subjective norm, and entrepreneurial intention. Entrepreneurial intention is the direct factor of entrepreneurial behavior and is affected by attitude, subjective standard, and perceived behavioral control (Szerb et al., 2019). Attitude is the degree of individual preference for entrepreneurial behavior, forming a comprehensive evaluation; the subjective standard is a kind of subjective cognition and is the individual subjective judgment of pressure and environment; perceived behavioral control is a subjective evaluation of the difficulty of entrepreneurial behavior, and entrepreneurial intention is an individual’s attitude toward entrepreneurial behavior and is the subjective criteria affecting personal judgment and behavior control (Vdovichenko et al., 2020).

**FIGURE 1:**
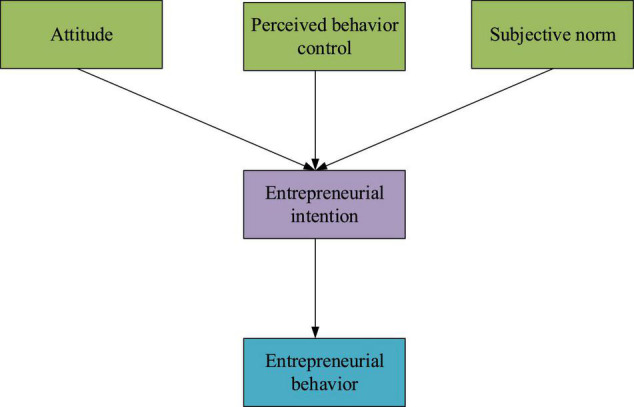
Theoretical model of planned behavior.

To sum up, environmental factors may affect behavior consciousness and behavior. Based on the research results of entrepreneurial consciousness, environmental factors are divided into three dimensions: family environment, the school and social environment, and the entrepreneurial environment. Up to now, much research has been conducted on the school environment and social environment, mainly focusing on entrepreneurship education, entrepreneurship policy, and entrepreneurial atmosphere (Andrew, 2021). Here, the family environment affecting the entrepreneurial consciousness of piano students in CAU includes family education atmosphere, family economic conditions, and parents’ objective situation; the school environment includes entrepreneurial practice, entrepreneurial courses, entrepreneurial base, and school atmosphere construction; meanwhile, the social environment includes enterprise support, entrepreneurial policy, school-enterprise cooperation, and government support. Creatively, some hypotheses are proposed involving the content of the entrepreneurial environment to demonstrate the relationship between various dimensions of the entrepreneurial environment and entrepreneurial consciousness (Ataei et al., 2020). The theoretical model of planned behavior is shown in Figure 1.

#### Personal Background Factors of Piano Majors in Colleges and Universities

According to the previous relevant literature, the views on the impact of personal background on college students’ entrepreneurial consciousness differ significantly, caused by students’ different work experiences and gender differences. Here, students’ entrepreneurial background is set as the research variable. Their personal background is set as the control variable, based upon which the influence of students’ background on entrepreneurial consciousness is analyzed among Piano Majors in CAU (Newman et al., 2019).

### Questionnaire Design

Then, the QS is designed according to the research on the theory of entrepreneurial PSYQ and students’ entrepreneurial PSYQ at the present state to accurately grasp the current situation of entrepreneurial PSYQ of college Piano Majors. Afterward, the present work excavates the existing problems and explores the countermeasures. Specifically, the QS is divided into two parts: the first part is the personal background information, including gender, grade, growth area, parents’ education level, cadres, participation in entrepreneurial practice, and secondary education. The second part measures achievement motivation, entrepreneurial quality, entrepreneurial interest, entrepreneurial attitude, entrepreneurial consciousness, interpersonal network, and entrepreneurial environment of Science and Engineering college students. Based on these variables and the previous relevant literature, the QS item is designed for each variable. The items in the QS are scored by the Likert-five-grade method (Utami, 2019).

(1) Metric of entrepreneurial attitude

The entrepreneurial attitude scale is mainly designed from three aspects: entrepreneurial possibility, entrepreneurial plan, and entrepreneurial idea containing six relevant metric items.

(2) Metric of achievement motivation

The achievement motivation scale is designed by considering the characteristics of college Piano Majors, including material pursuit, social contribution, and self-worth realization, with six related metric items altogether.

(3) Metric of entrepreneurial quality

Combined with the existing metric scale and the characteristics of college Piano Majors in Colleges, the entrepreneurial quality scale is designed from innovative thinking, risk challenges, and overcoming difficulties, with six metric items.

(4) Metric of entrepreneurial interest

With six metric items, the entrepreneurial interest scale is designed from inquisitiveness, initiative, and curiosity.

(5) Metric of entrepreneurial consciousness

According to the definition of entrepreneurship conscious and college Piano Majors and the existing metric scale, the entrepreneurship consciousness scale is designed with six relevant metric items.

(6) Metric of interpersonal network

By combining the research of College Students’ social network with the characteristics of college Piano Majors, the interpersonal network scale is designed from three aspects: individual, group, and relationship network, covering the interpersonal relationship strength, heterogeneity, and capacity. Overall, it contains six relevant metric items.

(7) Metric of entrepreneurial environment

According to the particularity of the research subject and the previous related research, this paper designs entrepreneurial environment scale from three aspects: school education, national policies, and family atmosphere. Because college Piano Majors live on campus most of the time, they think more about IEE. By analyzing the existing measurement scales related to college IEE, this paper designs the IEE topic into the entrepreneurial environment scale for this investigation, containing six metric items altogether.

(8) Metric of personal background

The college Piano Majors’ personal background is measured from the basic and entrepreneurial backgrounds. The former is taken as the control for the entrepreneurial PSYQ of college Piano Majors. Specifically, the control variables involve gender, grade, only child or not, served as student cadres or not since childhood. The entrepreneurial background is measured from three aspects: economy, influence, and education containing six relevant metric items.

Then, the SEM is constructed by taking the achievement motivation, entrepreneurial quality, entrepreneurial interest, interpersonal network, and entrepreneurial environment of college Piano Majors as exogenous variables, personal background as certain influencing factors, the entrepreneurial attitude of Science and Engineering students as intermediary variables, and entrepreneurial consciousness as outcome variables. The four variables of achievement motivation, entrepreneurial quality, interpersonal network, and entrepreneurial environment are second-order variables. Further, to streamline the model, the second-order dimensions of the four variables are packaged and calculated. Differently put, it averages all item scores in the second-order dimension, sums the scores of each topic under each dimension, and then divides the summation by the number of items, thereby scoring each dimension. Thus, the second-order dimension is converted into the first-order index. The simplified model does not reduce the parameter estimation accuracy while improving the model’s Goodness Of Fit (GOF) to a great extent.

### Selection of Respondents

The QS is designed based on Xi’an University of Finance and Economics and by recruiting the college Piano Majors through random sampling. Altogether, 509 QSs are distributed, with 483 valid QSs, an effective rate close to 94.9%. At the same time, QS method and direction communications with students are combined to explain students’ possible doubts on the spot, thereby further improving the QS effectiveness. Importantly, researchers have communicated with students from multiple perspectives during the survey. As a result, the QS results can truly reflect the real situation of students and can be used to investigate and research college piano talents.

## Data Statistical Analysis

### Reliability and Validity Analysis

A: Reliability Analysis

This paper obtains Cronbach’s α coefficients of 8 scales through analysis, as counted in Table 1.

**TABLE 1:** Statistics of Cronbach’s a coefficient of the scale.

Scale	A	B	C	D	E	F	G	H
Cronbach’s α	0.875	0.882	0.862	0.893	0.915	0.885	0.851	0.883

*A: entrepreneurial attitude; B: achievement motivation; C: entrepreneurial quality; D: entrepreneurial interest; E: Entrepreneurial consciousness; F: interpersonal network; G: entrepreneurial environment; H: personal experience.*

Normalized Cronbach’s α of eight scales are all greater than 0.8, indicating that the scale designed has high reliability and meets the reliability test standard. Cronbach’s α of entrepreneurial attitude metric, achievement motivation metric, entrepreneurial quality metric, entrepreneurial interest metric, entrepreneurial consciousness metric, interpersonal network metric, entrepreneurial environment metric, and personal background metric are 0.875, 0.882, 0.862, 0.893, 0.915, 0.885, 0.851, and 0.883, respectively. It has good reliability, stable and reliable measurement.

B: Scale validity analysis

**FIGURE 2:**
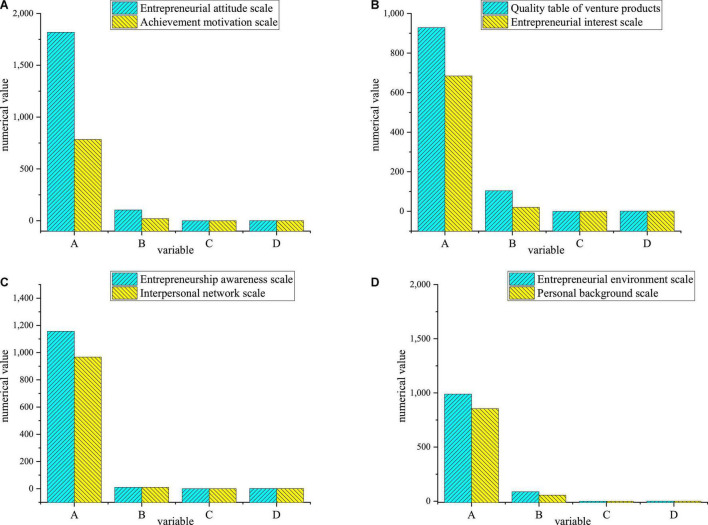
Statistics of validity analysis results [**(A)** entrepreneurial attitude scale and achievement motivation scale; **(B)** entrepreneurial quality scale and entrepreneurial interest scale; **(C)** entrepreneurial consciousness scale and interpersonal network scale; **(D)** entrepreneurial environment scale and personal background scale; A: approximate chi-square; B: Degree of Freedom (DOF); C: significance; D: statistical test].

**FIGURE 3:**
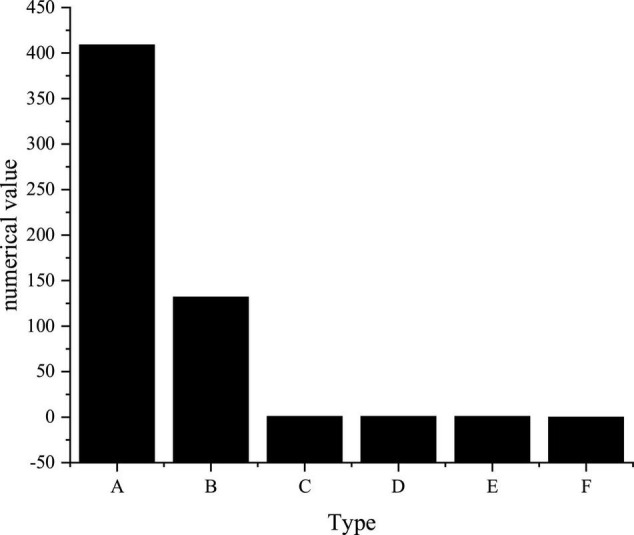
Fit test [A: λ^2^; B: Df; C: GOF D: Comparative Fit Index (CFI); E: Model fitting index; F: Root Mean Square Error of Approximation (RMSEA)].

Figure 2 analyzes the validity of each scale:

As in Figure 2, the Kaiser Meyer Olkin (KMO) values of the entrepreneurial attitude scale, achievement motivation scale, entrepreneurial product quality scale, entrepreneurial interest scale, entrepreneurial consciousness scale, interpersonal network scale, entrepreneurial environment scale, and personal background scale are all above 0.8. Thus, these eight scales are very suitable for structural validity analysis. The KMO values of all scales are greater than 0.7 or about 0.7, and the results of the Bartlett spherical test are significant (the significant values are 0.000 < 0.01), indicating that the correlation between variables is solid and independent of each other. It can be proved that the data results of the QS are suitable for structural validity analysis. Figure 3 gives fitting index test results.

As in Figure 3, the fitting indexes of the four indexes have reached an acceptable or better level. The four main variables have good validity so that the subsequent hypothesis test can be carried out. Apparently, most of the fitting indexes of the four scales meet the test requirements, and some indexes have shown excellent value, indicating that the model and the data fit perfectly.

### Statistics of Different Types of Data

**FIGURE 4:**
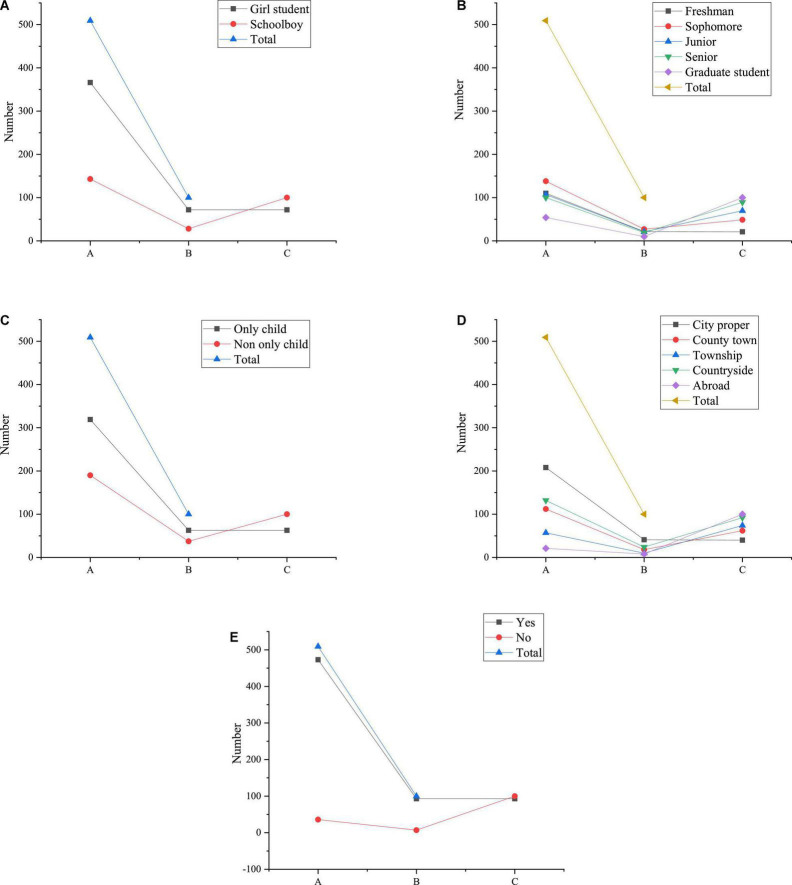
Classified statistics of sample data [**(A)** Gender composition **(B)** Grade composition **(C)** The only child composition **(D)** Growth environment composition **(E)** The composition of student cadres A: Frequency statistics B: Percentage (%) C: Cumulative percentage (%)].

Figure 4 illustrates the classified statistics of gender, grade, only-child-or-not, growth environment, and student-cadre-or-not in the piano talent sample of CAU.

Figure 4 reveals that the proportion of girls and boys in the sample is 72.9% and 27.1%, respectively, which is in line with the proportion of male and female piano students in CAU. Therefore, the sample must be sufficiently representative. Besides, according to the data statistics of different grades, the piano students in the first-year account for 21.6%, the piano students in sophomore year account for 27.1%, the piano students in junior year account for 21.0%, and the piano students in senior year account for 19.6%. The proportion of four grades of undergraduate students is basically equal, and the master students account for about 10.7% of the total samples. Obviously, the proportion of postgraduates is relatively low; this is because there are relatively few postgraduates majoring in piano at this stage, which is in line with the actual situation of CAU. At the same time, in the statistics of whether the sample is an only child or not, the proportion of only children and non-only children is 62.7% and 37.3%, respectively. According to the statistics of life backgrounds during their middle school years, more students live in urban areas than in other environments, with a proportion close to 40.9%. Compared with other living environments, the proportion of students living abroad is the least, accounting for nearly 10.2%. Lastly, the statistics show that nearly 92.1% of the college Piano Majors have served as student cadres. Therefore, the selection of samples in the QS is targeted and representative

### Descriptive Statistical Analysis of Samples

**FIGURE 5:**
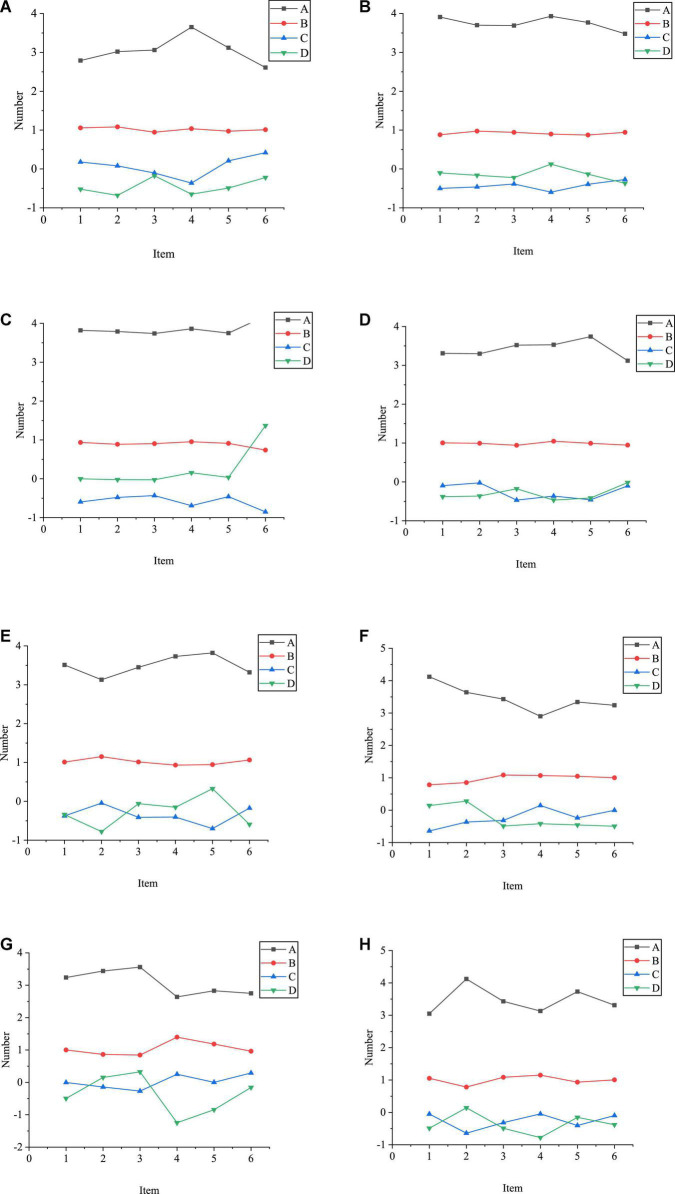
Results of sample descriptive statistical analysis [**(A)** Measurement items of entrepreneurial attitude variables; **(B)** Measurement items of achievement motivation variables; **(C)** Measurement items of entrepreneurial quality variables; **(D)** Measurement items of entrepreneurial variables; **(E)** Measurement items of entrepreneurial consciousness variables; **(F)** Measurement items of interpersonal network variables; **(G)** Measurement items of entrepreneurial environment variables; **(H)** Measurement items of personal background variables. A: Mean value; B: Standard deviation; C: Skewness; D: Kurtosis].

Figure 5 demonstrates the results of descriptive statistical analysis of the sample.

The sample descriptive statistics in Figure 3 corroborate that the entrepreneurial quality in Figure 3C is a controllable variable. Although the skewness and kurtosis of the Figure do not meet the requirements, it will not affect the research results. The changes of all variables in other Figures show a normal distribution trend. The absolute kurtosis of each variable is smaller than 10, and the absolute skewness is lower than 3. Therefore, the above data meet the needs of the designed experiment.

### The Factor Differential Test on the Cultivation of Entrepreneurial Psychological Quality

**FIGURE 6:**
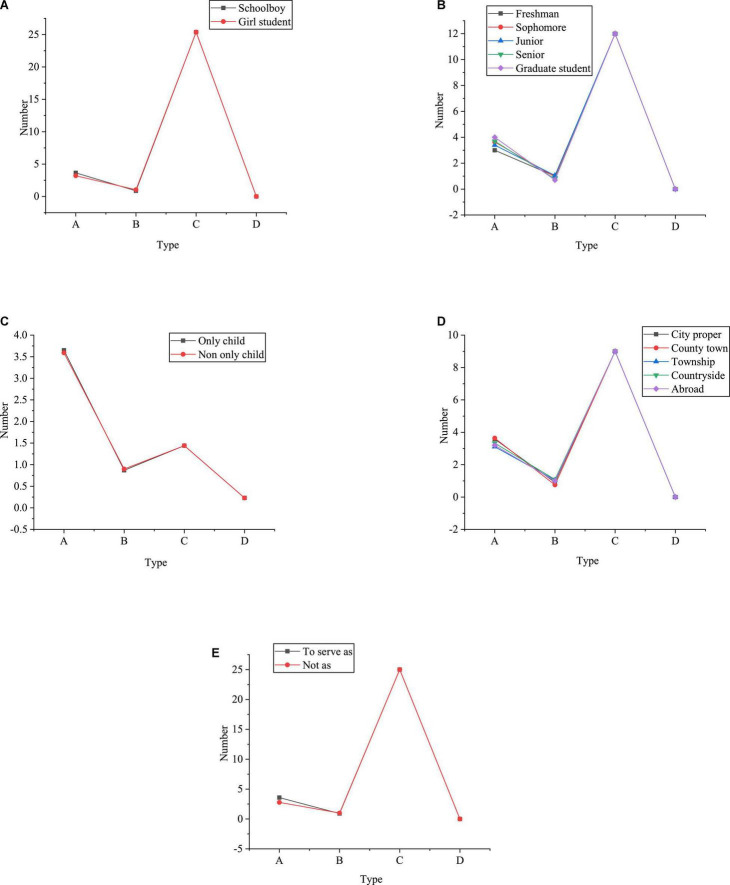
The differential test of entrepreneurial PSYQ cultivation [**(A-E)** represents the differential test of entrepreneurial PSYQ cultivation of college piano talents with different genders, different grades, only-child-or-not, growth environment, and student-cadres-or-not, respectively. A: Mean. B: Standard deviation. C: F-value. D: P-value].

Figure 6 shows the differential test of entrepreneurial PSYQ training.

Statistics from Figure 5 reveal significant differences between male and female students in cultivating entrepreneurial PSYQ, namely, *p* < 0.01. Boys majoring in piano have stronger entrepreneurial PSYQ than girls. Moreover, there are significant differences in the cultivation of entrepreneurial PSYQ among college Piano Majors of different grades, namely, *P* < 0.01. Under comparative analysis, the entrepreneurial PSYQ of first-year students is the lowest, the entrepreneurial PSYQ of sophomores and junior students is almost the same, and the entrepreneurial PSYQ of senior students is much higher than that of other undergraduates. However, the entrepreneurial PSYQ of postgraduates is higher than that of undergraduates mainly because most postgraduates have experienced interviews, work, and different experiences. There is no significant difference in entrepreneurial PSYQ between college Piano Majors in terms of only-child-or-not, namely, *P* > 0.1. By contrast, students who have grown up in different environments have shown significant differences in entrepreneurial PSYQ, namely, *p* < 0.01. Under statistical analysis, college Piano Majors who have grown up in urban areas show the strongest entrepreneurial desire and entrepreneurial PSYQ. Therefore, t different living environments will affect the entrepreneurial PSYQ of college Piano Majors. Additionally, having served as student cadres also has a particular impact on students’ entrepreneurial PSYQ, namely, *p* < 0.01. Statistics show that students who have served as student cadres during school are more intense in entrepreneurial PSYQ than those not.

### Structural Equation Modeling of Factors of College Students’ Entrepreneurial Consciousness

**FIGURE 7:**
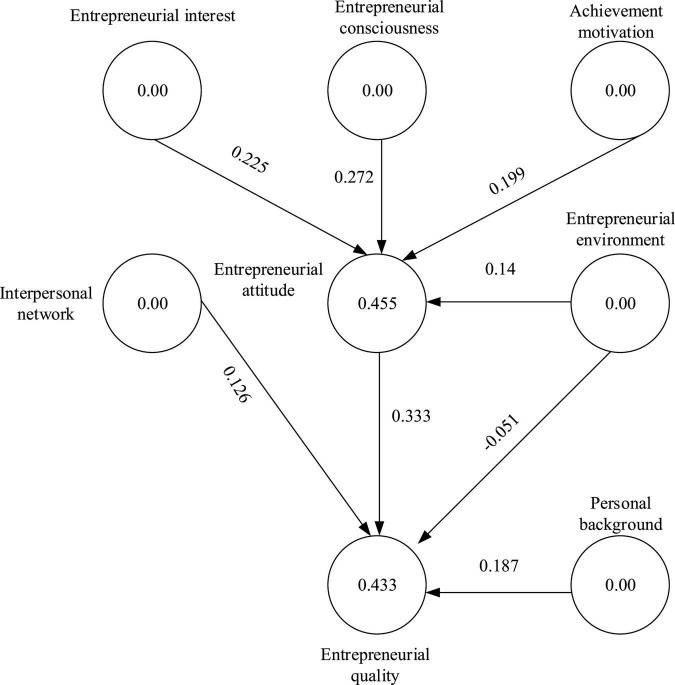
Statistical results of SEM of factors of entrepreneurial PSYQ training of college piano talents.

Figure 7 displays the SEM statistics of the factors of cultivating entrepreneurial PSYQ of college piano talents.

(I) There is a significant positive correlation between college students’ achievement motivation and entrepreneurial attitude; the model path is 0.199, *P* < 0.01. (II) Entrepreneurial consciousness has a significant positive correlation with entrepreneurial attitude; according to the significance test results of the model path coefficient, the model path value is 0.272, *P* < 0.01. (III) Entrepreneurial interest has a significant positive correlation with an entrepreneurial attitude, and the model path value is 0.225, *P* < 0.01. (IV) Entrepreneurial attitude has a significant mediating effect on entrepreneurial PSYQ, and its model path value is 0.333, *P* < 0.01. (V) There is a significant positive correlation between interpersonal network and entrepreneurial attitude, and the model path value is 0.14, *P* < 0.01. (VI) The interpersonal network has no direct impact on entrepreneurial PSYQ. (VII) The entrepreneurial environment has a significant positive correlation with entrepreneurial PSYQ, and its model path value is 0.126, *P* < 0.05. (VIII) There is a significant positive correlation between personal entrepreneurial background and entrepreneurial PSYQ, and the model path value is 0.187, *P* < 0.01. To sum up, innovative thinking, risk challenges, and anti-adversity resilience are the psychological qualities that most affect the entrepreneurial consciousness of college Piano Majors. Therefore, schools and families should focus on cultivating these three psychological qualities of Piano Majors to help them better start a business.

## Discussion

Liguori et al. (2018) researched how self-efficacy shaped entrepreneurial intention and debated the role of domain-specific self-efficacy and generalized self-efficacy. They proved that personal input (generalized self-efficacy, gender, and minority status) and environment/background input (previous work experience, entrepreneurial experience, and family business contact) significantly affected entrepreneurial self-efficacy and entrepreneurial outcome expectation. They also analyzed the influence of external environment and teaching factors on college students’ entrepreneurial consciousness rather than students’ perspectives (Liguori et al., 2018). The most prominent feature of the present work is investigated from students’ perspectives. Data analysis results indicate that IEE has the greatest impact on the entrepreneurial PSYQ of Piano Majors, followed by the impact on entrepreneurial ability, and the impact on the entrepreneurial quality is the least. Quality is regarded as a mental and psychological structure and a congenital tendency, making individuals respond to stimuli in a relatively consistent, stable, and lasting way. Therefore, there is a need to strengthen the construction and promotion of IEE courses. Because college IEE is one of the most important ways to improve the cultivation of entrepreneurial PSYQ of Piano Majors, CAU should expand the breadth and depth of IEE courses and promote IEE. Its core is to spread, promote, and practice the spiritual essence of IE, namely, an entrepreneurial quality with the courage to explore and innovate. Overall, IEE should guide students to understand and think about entrepreneurship from an objective perspective, cultivate their innovative PSYQ, and apply the innovative PSYQ to a wider range of social practices.

## Conclusion

At present, the domestic economic growth is slowing down and, thus, its pulling effect on employment has been weakening. Such an employment difficulty has been aggravated by college students’ limited overall quality, including insufficient work experience, so the employment situation is not that optimistic. Under such social background, how to improve college students’ entrepreneurial PSYQ is worth in-depth research and is extremely important for national development. Accordingly, this paper studies the cultivation of piano talents and entrepreneurial PSYQ in CAU through QS design that statistically analyzes the relevant data of college Piano Majors. Then, the influencing factors of entrepreneurial PSYQ cultivation are modeled, processed, and examined using the SEM. The results corroborate that seven factors affect the cultivation of entrepreneurial PSYQ of college Piano Majors, and their correlation with PSYQ is positive. In contrast, the interpersonal network will not affect the cultivation of students’ entrepreneurial PSYQ. This research will further help CAU cultivate the entrepreneurial PSYQ of Piano Majors and help them grow into excellent social talents.

Nevertheless, there are some limitations. For example, the selected sample is from a university in Xi’an. Compared with universities in coastal areas, the outcome based on a single in-land higher institution might not be that convincing. Moreover, the research findings might somehow get biased from the influence of Internet technology. Secondly, due to the limited time and energy, the present work has chosen to use the horizontal research method of group survey, with no involvement of any longitudinal research method based on time or event process (such as changes from college students’ enrollment to graduation, before and after participating in specific courses or activities). Therefore, in terms of IEE, future research will further tap the connotation and extension of relevant variables and measure them from multiple levels. The follow-up study expects to expand the number and scope of samples to various CAU across China to enrich collected data and generalize the research results.

## Data Availability Statement

The raw data supporting the conclusions of this article will be made available by the authors, without undue reservation.

## Ethics Statement

The studies involving human participants were reviewed and approved by Shandong Normal University Ethics Committee. The patients/participants provided their written informed consent to participate in this study. Written informed consent was obtained from the individual(s) for the publication of any potentially identifiable images or data included in this article.

## Author Contributions

The author confirms being the sole contributor of this work and has approved it for publication.

## Conflict of Interest

The author declares that the research was conducted in the absence of any commercial or financial relationships that could be construed as a potential conflict of interest.

## Publisher’s Note

All claims expressed in this article are solely those of the authors and do not necessarily represent those of their affiliated organizations, or those of the publisher, the editors and the reviewers. Any product that may be evaluated in this article, or claim that may be made by its manufacturer, is not guaranteed or endorsed by the publisher.
